# Social Media Behavior and Emotional Evolution during Emergency Events

**DOI:** 10.3390/healthcare9091109

**Published:** 2021-08-27

**Authors:** Mingyun Gu, Haixiang Guo, Jun Zhuang

**Affiliations:** 1 College of Economics and Management, China University of Geosciences, Wuhan 430074, China; gmingyun@gmail.com; 2 Research Center for Digital Business Management, China University of Geosciences, Wuhan 430074, China; 3 Mineral Resource Strategy and Policy Research Center, China University of Geosciences, Wuhan 430074, China; 4 Department of Industrial and Systems Engineering, University at Buffalo, SUNY 317 Bell Hall, Buffalo, NY 14260, USA

**Keywords:** social media analysis, online public sentiment, sentiment evolution, emergency

## Abstract

Online social networks have recently become a vital source for emergency event news and the consequent venting of emotions. However, knowledge on what drives user emotion and behavioral responses to emergency event developments are still limited. Therefore, unlike previous studies that have only explored trending themes and public sentiment in social media, this study sought to develop a holistic framework to assess the impact of emergency developments on emotions and behavior by exploring the evolution of trending themes and public sentiments in social media posts as a focal event developed. By examining the event timelines and the associated hashtags on the popular Chinese social media site Sina-Weibo, the 2019 Wuxi viaduct collapse accident was taken as the research object and the event timeline and the Sina-Weibo tagging function focused on to analyze the behaviors and emotional changes in the social media users and elucidate the correlations. It can conclude that: (i) There were some social media rules being adhered to and that new focused news from the same event impacted user behavior and the popularity of previous thematic discussions. (ii) While the most critical function for users appeared to express their emotions, the user foci changed when recent focus news emerged. (iii) As the news of the collapse deepened, the change in user sentiment was found to be positively correlated with the information released by personal-authentication accounts. This research provides a new perspective on the extraction of information from social media platforms in emergencies and social-emotional transmission rules.

## 1. Introduction

Social media is widely followed by billions of people because of its focus on human activities [1], with the information generated and disseminated now an essential part of many people’s everyday lives [2]. However, even though much of this information is created and disseminated by ordinary users, many people rely on their social media feeds for news about trending events [3,4,5,6], public opinion [7,8,9], market analyses [10,11,12] and influencers [13,14,15,16]. Since its launch in May 2009, Sina-Weibo has been the most popular microblogging platform in China, and has become the most common global real-time information network. Users create status messages and give their opinions on wide range of topics [17]. Sina-Weibo also allows users to interact with others using tools such as mention (@, @username, which means that the message sender is willing to share this post with the user/users mentioned, retweet, which means the users are usually forwarding messages they are interested in from other users, hashtag (##, contents between## refer to a specific topic), and like, which is when users agree with messages posted by other users [18].

Consequently, social media has been researched and analyzed from different angles based on these variable social media data characteristics. For example, Zhao et al. used personal information [19], Sina-Weibo content and social relations to analyze Sina-Weibo user features based on interest detection, and taking the 2015 Tianjin explosion as an example, Wu studied the dynamics of the Sina-Weibo user challenges to the official discourse [20]. As the data quantities and the web text features increase, ordinary algorithms are no longer able to fulfil research needs, which means that classifiers are now being integrated into analyses [21,22,23]. Therefore, this study also combined social media data and integration algorithms. Goel et al. used structured virality to analyze a unique data set of one billion tweets to assess the mechanisms leading to increases in epidemic event communications through broadcast and viral mechanisms [24], Khatua et al. extracted meaningful semantic relationships from a relatively small domain-specific Twitter input corpus [25], and Hasan et al. proposed an event detection system called TwitterNews+ to detect major and minor newsworthy events in real time [26]. Influenced by these innovations, this study also divided the corpus selection and hot-spot data selection. In addition, scholars are also keen to study the role of rumors and misinformation during emergencies [27]. The study of rumors and misinformation plays a key role in making better use of social media during emergencies and this inspired Falavarjani et al. to conduct a correlation analysis of the online and offline behaviors of the social media users [28]. 

As well as identifying user characteristics and mining information transmission modes, user emotions have also attracted research attention, with the sentiments being classified using supervised, unsupervised, and semi-supervised machine-learning techniques [24,29,30,31]. Because of the progress made in deep learning, deep learning was also applied in this study to elucidate the emotions in the relevant posts. We studied the emotional expression under different topics and compare the differences between them. Through our comparative analysis, we get the differences of emotional expression under different topics during emergencies. The study results expand existing research on social media emotions. This paper used the BiLSTM algorithm to identify the emotional tendency of the of the captured data.

Past research has proven the effectiveness of using machine learning to analyze emergencies; however, while most research has selected data related to accidents, these analyses have been too concentrated on a universal theme, with little further analysis of the data sources, that is, the multiple focus events emerging from the same accident/emergency have not been considered even though these events may have different effects on user behavior. Therefore, in this study, the 2019 Wuxi viaduct collapse accident was taken as the main research object and the various focus events emerging as the accident discussions developed also evaluated to assess how the development of emergencies affect the evolution of user sentiment and posting behaviors.

The remainder of this paper is organized as follows. Section 2 reviews related work on mining Sina-Weibo data and the use of social media during emergencies and crises, Section 3 introduces the data sources and methodology, Section 4 discusses the experimental results, and the results of the topic and sentiment analyses related to the 2019 Wuxi viaduct collapse, and Section 5 concludes this paper and discusses future research directions.

## 2. Related Work

Related work in two main areas is examined in this section: mining Sina-Weibo data research, and the use of social media in emergencies and crises.

### 2.1. Mining Sina-Weibo Data

There has been significant research focused on micro-blog data. For example, Liu et al. developed a new method that determined the probabilities for the different fields a user might be interested in and then calculated the probabilities of user interest in new topic [32], and Zhao et al. proposed a method involving microblog filtering, sentiment analysis, and stock market predictions based on an emotional analysis of Sina-Weibo user data [19]. Based on the Sina-Weibo data set, Xu et al. improved user recommendations by extracting social topics and topics of interest from users in one-way social networks [33], and Wang et al. proposed a business rule framework plus (BRF++) model to fully mine the relationship between users and predict their forwarding behaviors [34].

Taking the 2019 Wuxi viaduct collapse as an example, this paper collected Sina-Weibo microblog data from all dimensions, such as user information, release time, and content, under eight themes, since users can choose whether to display the location when publishing microblogs, we cannot get all the accurate location information of microblogs, so the index of location was not considered in this study. Thus, we analyzed the data and developed a social media data framework to assess the impact of public emergencies.

### 2.2. Use of Social Media during Emergencies and Crises

Recent research has proposed that social media data streams could be used to mine actionable data for emergency response and rescue operations because of the significant increase in social media use during emergencies and crises [35]. Alexander outlined seven uses of social media posts: listening to public debate [36]; monitoring situations; extending emergency response and management; crowd-sourcing and collaborative development; creating social cohesion; furthering causes; and enhancing research. Obtained data have generally been divided into four dimensions; time, space, content and network; all of which respectively correspond to the Twitter metadata fields of coordinates/place/location, timestamp, text and retweet [37,38]. This division was also applied in this research. 

Time. Time series analyses have found that as a crisis moves from the prodromal to the acute phase, information becomes less common and information on specific remedial actions is lacking [39]. Cozza et al. proposed social–spatio temporal analysis of tweets and as case study reported on Hurricane Isaac [40]. Pourebrahim et al. surveyed Twitter usage during 2012 Hurricane Sandy and found that Twitter was a very valuable source of disaster-related information, especially during power outages [41], Wang et al. used trend analyses to reveal the Sina-Weibo data emergency communication fluctuations based on the 2012 Beijing Rainstorm [2], and Fang et al. used a time evolution of social media activities to track the process of the 2016 Wuhan Rainstorm, confirmed that the time changes in the social media activities were consistent with the heavy rain process, and that the social media activities were significantly positively correlated with precipitation intensity [1]. 

Space. Public safety and emergency management requires semi-automatic tools that can quickly detect the type, scope, location, intensity, and impact of natural or man-made disasters and emergencies and give accurate and detailed situation reports [42]. For example, Middleton et al. developed a real-time crisis map platform to localize tweet content [43], Chae et al. developed an interactive visual spatiotemporal analysis and spatial decision support environment system to assist with evacuation planning and disaster management [44], De Albuquerque et al. found that the probability of flood-related information was much higher near severely flooded areas up to 10 km long during the River Elbe Flood of June 2013 in Germany [45], and Smith et al. proposed a real-time modeling framework that used data obtained only through social media to identify areas where flooding was likely to occur [46].

Content. To explore the general behavior of users during disaster events, research has sought to more efficiently use social media data to determine the needs of the public during the rescue process, to refute rumors, and to improve crisis management capabilities [47,48,49,50,51,52,53]. Public sentiment during emergencies has also been examined to identify the evolutions in public emotions and assist governments to positively adjust available information. These diverse public sentiments have been found to influence information dissemination through official media and the Government’s decision-making [54]. For example, Han and Wang found that the quantity of texts on Sina-Weibo changed over time, with different themes and emotions corresponding to different stages of an emergency [55].

Network. More and more scholars are committed to the research of information dissemination mechanism in social networks [56], and try to explore the relationship between nodes in the network [57], these results also provide suggestions for commercial marketing [58]. The visualization of social network analysis results can assist in inferring information transmission during disasters [59,60,61], with various indicators, such as intermediary centrality, intimacy and PageRank, being used to quantitatively detect network patterns [62]. For example, Wang and Zhuang studied the social media information distribution and coverage during the Hurricane Sandy disaster [63], and based on the Sina-Weibo reposting behaviors during the Yiliang Earthquake, L Li et al. developed a content-based multi-category Naïve-Bayesian classifier that divided the microblog posts into five categories, after which the transmission patterns of these five categories were characterized in different stages after the earthquake [64].

This paper used a classification method to collect data, divide the research focus, and obtain the data based on different research perspectives. Because previous research has been mainly focused on the analysis of the disaster event itself, the quantity of data collected was extensive. However, in this paper, the data collection first used the Sina-Weibo characteristics to identify the focused events at different event points, and then used different tags to complete the data acquisition and complete the accident timeline.

Previous research has also tended to focus on the role of emergencies in stimulating the social media network user activity, in recent years, some scholars are also paying attention to and studying the dissemination of misinformation and how it affects people’s opinions [27,65]. Therefore, this paper examined the data characteristics to elucidate the dissemination mode for new focused news over time, with a comparative analysis conducted to identify the rules. Past research has tended to only focus on singular events in the analysis of user emotions or has focused on improving the emotional judgment accuracy by developing better algorithms; however, few studies have combined the changes in the user emotions with the emergency development process or paid attention to the role of new news foci on the public opinion fermentation process. Therefore, this paper established a complete sentiment analysis timeline, which when combined with the topic modeling method allowed for an exploration of the driving role of the emergency development process on the changes in user emotion and behavior. Using the constructed research framework, this paper conducted a comparative analysis on the changes in the user emotions and behavior to reveal the change laws for the same and different themes in the transmission process over time.

## 3. Data and Methods

**Figure 1:**
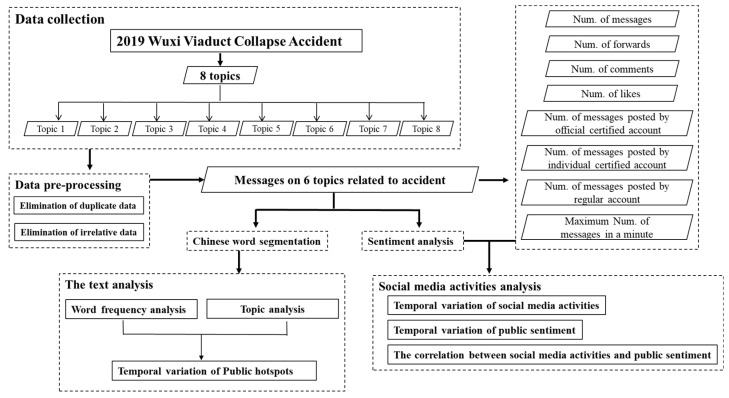
Social media disaster assessment framework and method used for the analysis of the “2019 Wuxi Viaduct Collapse”.

Figure 1 shows the social media disaster assessment framework and method used in this paper, for which there were four main parts: data collection; preprocessing; disaster process analysis; and disaster impact analysis. The framework was developed to use social media data to characterize the user behavior and the sentiment analysis.

### 3.1. Study Case Description

**Figure 2:**
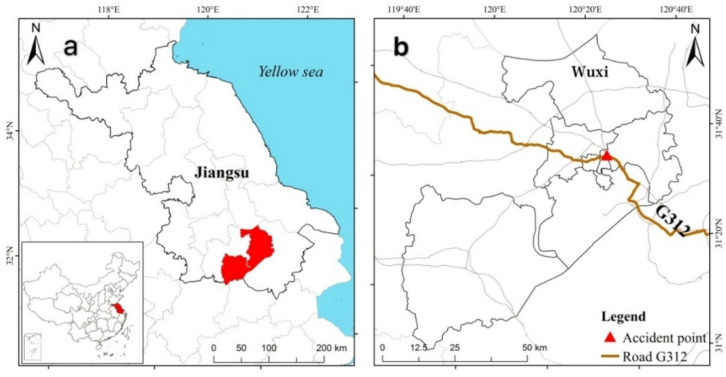
Location of Wuxi (**a**), G312 and accident site (**b**).

Wuxi, a prefecture-level city in Jiangsu province, is one of the central cities along the Yangtze River delta that has been approved by the state council as an important scenic tourism city (Figure 2a). On 10 October 2019, the K135 National Highway 312 viaduct in Xishan district, Wuxi, collapsed, killing three people and injuring two other and causing direct economic losses of 8.231 million CNY, all of which had a significant social impact. The collapse happened on the 312 national-road, which is one of the most important connection roads and therefore carries large volumes of transit and freight transportation traffic. The intensive construction along the land and in the peripheral areas of the city, and especially on the north ring road of the 312 national highway and S342 provincial highway caused severe transport congestion in Wuxi (Figure 2b).

**Figure 3:**
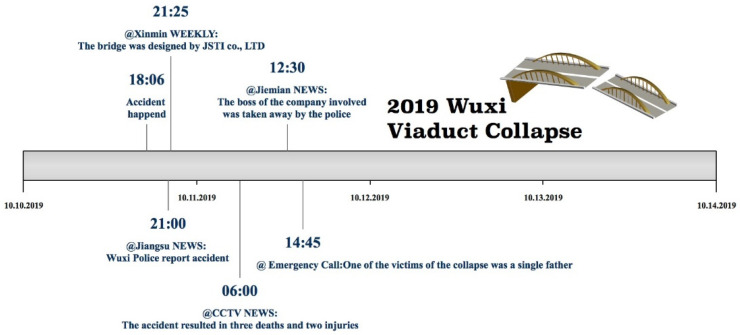
Important time nodes and developments in “2019 Wuxi Viaduct Collapse”.

Some important time nodes and developments in this event were as follows: (1) Around 18:10 on the evening of 10 October 2019, the K135 Bridge deck of the 312 National Road in Wuxi Xishan district, collapsed onto three cars underneath the bridge blocking traffic; (2) at 21:00 on 10 October 2019, the Wuxi police reported the viaduct collapse and the initial situation; (3) at around 21:00 on 10 October 2019, the media reported that the bridge design company had been JSTI Co., Ltd.; (4) at 6:00 am on 11 October 2019, information released by the accident rescue headquarters stated that an expert team from the Ministry of Transport had rushed to the scene to guide the accident investigation and an accident investigation team had been established in Wuxi—after the preliminary analysis, the viaduct collapse was claimed to be caused by an overload and resulted in three deaths and two injuries; (5) on 11 October 2019, the director of Wuxi Success Transportation Co., Ltd. was arrested, the company shuttered and the employees dismissed; (6) at 14:45 on 11 October 2019, an official social account on Weibo, *@Emergency Call*, revealed that one of the victims was a single father. (Figure 3).

### 3.2. Data and Pre-Processing

#### 3.2.1. Data

People using Sina-Weibo, which is one of the most popular social media sites in China, can release news, get information, participate in discussions, and express their emotions. The Sina-Weibo annual report stated that they had reached 516 million monthly active users in 2019, 94% of whom had mobile accounts. With 222 million daily active users, the endless data stream makes Sina-Weibo an important data source for social media research.

In Sina-Weibo, users can post microblogs under a certain theme, with different themes corresponding to different discussion interests. As shown in the accident development timeline in Figure 3, the important events occurring at the different time nodes were taken as the key objects for the data collection. Leveraging the hashtag function in Sina-Weibo, eight themes were found to correspond to six events. Using the Sina-Weibo API, all 65306 Sina-Weibo posts under all themes from 18:00 on 10 October 2019 to 24:00 on 13 October 2019, were captured, as follows: 1. # A viaduct collapsed in Wuxi, Jiangsu province #, 2. # A viaduct collapsed in Wuxi #, 3. # A bridge collapsed in Wuxi #, 4. #Wuxi police reported viaduct collapse#, 5. # The viaduct was designed by JSTI Co., Ltd.#, 6. # 3 dead, 2 injured in Wuxi Viaduct Collapse #, 7. # The boss of Wuxi Success Transportation Co., Ltd., the vehicle’s owner, was taken away by police #, 8.# One of the victims was a single father #.

#### 3.2.2. Data Pre-Processing

Data pre-processing involved the preliminary cleaning and collation of the collected data. Data were collected for each message from multiple dimensions, such as username, microblog content, release time, and release tool, and the user type, message content, number of messages, number of forwards, number of comments, number of likes, and release time. As it was found that Themes 1, 2 and 3 had almost the same focus except for the different theme names, these were combined into one theme: #Wuxi Viaduct Collapse in Jiangsu province#. When duplicate data, advertising data and other data irrelevant to the study were eliminated from the original data, there were six main themes, 59,263 messages, 288,660 forwards, 540,421 comments, and 6,186,639 likes; the specific data for which is shown in Table 1. The six themes were: 1. # Wuxi Viaduct Collapse in Jiangsu province #, 2. #Wuxi police reported viaduct collapse#, 3. # The viaduct was designed by JSTI Co., Ltd.#, 4. # 3 dead, 2 injured in Wuxi Viaduct Collapse #, 5. # The boss of Wuxi Success Transportation Co., Ltd., the vehicle’s owner, was taken away by police #, 6. # One of the victims was a single father #. The remainder of this paper uses the Theme Numbers 1 to 6 to represent the each these themes.

### 3.3. Method

#### 3.3.1. Text Analysis

(1) Word Segmentation

Words frequently utilized within social media usually imply the foci. As the messages collected from Sina Weibo were in Chinese, word segmentation was necessary to regroup and synthesize the word sequences based on certain specifications. This study used the “Jieba” (https://github.com/fxsjy/jieba, accessed on 18 December 2019) word segmentation package to segment the text in the messages, which is one of the most widely used Chinese word segmentation tools. By constructing a user dictionary containing keywords related to “2019 Wuxi Viaduct Collapsed Accident”, the words were effectively segmented, after which words such as “me”, “you”, “ah” and punctuation were deleted. As part of the word segmentation process, “Jieba.loads” loads a custom dictionary, and then “Jieba” prioritizes the word segmentation based on the phrases in the dictionary, segments (parts-of-speech) based on the dependent grammar, uses “Jieba.cut” to segment the text, and finally uses the counter in the collection function to count the word segmentation data and determine word frequencies. In this study, word segmentation was performed in two ways:

First, use the ROST content mining software (Wuhan University, Wuhan, China, hereinafter referred to as ROSTCM6 software) developed and improved by the virtual learning team of Wuhan University for word segmentation. First, convert the preprocessed data file into a txt file, and then use the “word segmentation” function of the “functional analysis” in ROSTCM6 to segment the text content.

Second, use Pycharm (JetBrains, Prague, Czech Republic) software and the Jieba package to segment the data, you need to manually convert the txt source file into UTF-8 format, otherwise it will report a Chinese encoding error. Before word segmentation, the text needs to be processed to remove numbers, letters and special symbols. This can be achieved by using python’s own string and re modules. The string module is used to process string operations, and the re module is used to process regular expressions.

(2) The LDA Model

Latent Dirichlet allocation (LDA), which was first proposed by [66] and subsequently improved by [67], is an unsupervised Bayesian probability topic generation model that has three “document-topic-word” layers that can automatically learn and infer topics embedded in a given corpus without the need for manual data annotation. Therefore, this paper used the LDA model to extract the Weibo user topics that emerged during the Wuxi viaduct collapse event. In Section 4.2, the experimental results of LDA model in this paper are explained in detail.

#### 3.3.2. Sentiment Analysis

The bi-directional long short-term memory (BiLSTM) algorithm [68] was used to analyze the user sentiments. Building upon long short-term memory (LSTM) [69,70], a recurrent neural network was designed to model the temporal data in the likes on the texts, with the BiLSTM synthesizing the two LSTMs to learn each token in a textual sequence based on both the past and future contexts of the token. Therefore, the BiLSTM, which has proven to be effective for emotion analysis, was able to capture the two-way semantic dependencies.

In this paper, the BiLSTM algorithm was used to identify the emotional tendency of the text, and the microblog text data were divided into positive, negative, and neutral emotion tags for user emotion research.

## 4. Results and Discussion

### 4.1. Temporal Variation of Social Media Activities

**Figure 4:**
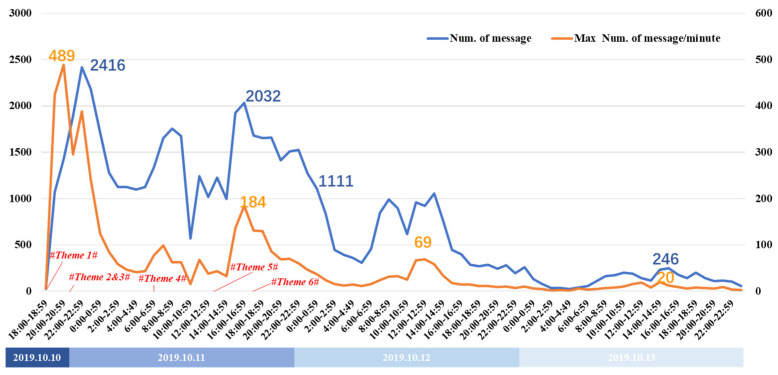
Trends for the number of messages and the maximum number of messages per minute.

The Weibo data for the six different themes was initially collected for 5 days after the accident; however, as the data quantity dropped sharply from the fourth day, data were collected from 6 p.m. on 10 October 2019 to 12 p.m. on 13 October 2019 for the analysis. The two curves in Figure 4 show the hourly changes in the number of messages and the maximum number of messages per minute. The maximum number of messages per minute was taken as the enthusiasm being shown to participate in the discussion. While messages may be the result of multiple postings by the same user, the maximum number of messages per minute indicates the popularity of participating in the discussion. It can be seen that the trends in the two curves were almost the same, which basically proved that in this event, the change in the microblog numbers reflected the users’ participation in the theme discussion and was the basis for the subsequent data analysis.

**Figure 5:**
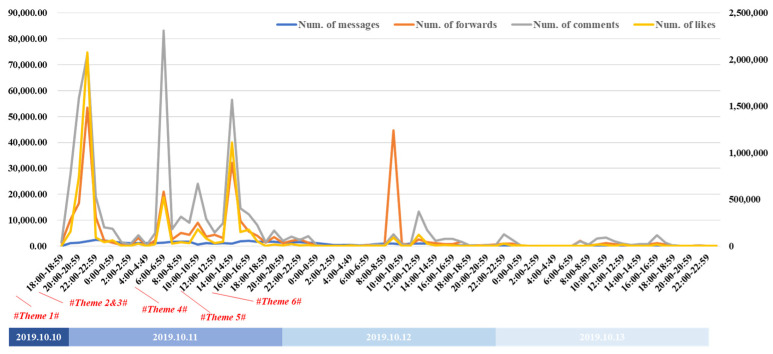
Number of messages/forwards/comments/likes related to the accident.

**Figure 6:**
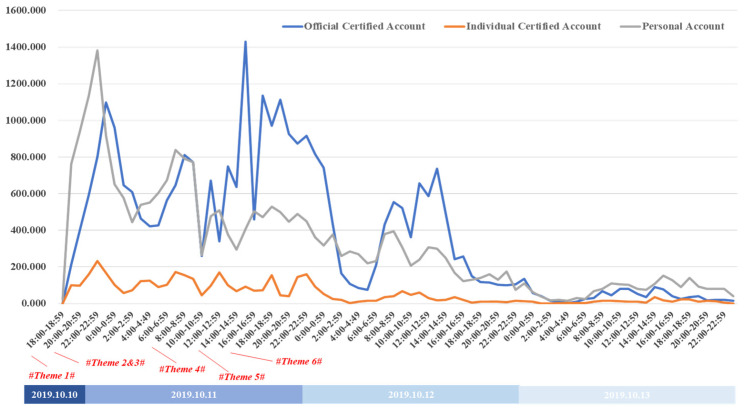
Number of messages posted by the different user groups.

Figure 5 shows the changes in the number of forwards/comments/likes, with the number of likes being higher than the forwards or comments, as it is the simplest action. These three metrics revealed how the users participated in the accident discussions, with the trend changes being basically the same. The similarities in the trends show that the social media activities had obvious temporal characteristics, with the total number of microblogs when combined with the temporal characteristics being similar to the users’ work schedules. The discussions continued until around 11 o’clock in the evening, with the other discussion peaks the following days being early in the morning, around the lunch break, and straight after work, as shown in Figure 6, which seemed to indicate that the users did not change their daily schedules to discuss this emergency.

The Sina-Weibo platform has three main types of users: official authentication accounts, such as official media, enterprises, and institutions; personal authentication accounts, such as actors, athletes, and entrepreneurs; and personal accounts for ordinary users, which are the greater in number. To understand the role of these three user types after the accident, Figure 6 was drawn up, which shows that when the accident occurred, the individual user responses were the fastest because they did not need to be particularly careful about their words; however, as official users do not represent individuals, their actions are not as rapid. One day after the incident, the number of officially authenticated accounts prevailed to report, discuss, and quash rumors about the incident. Compared with the other two types of accounts, the official authentication account had a stronger event continuity. Because of the relatively smaller number of users, the personal authentication account was small. However, the change trends in all three were similar.

**Figure 7:**
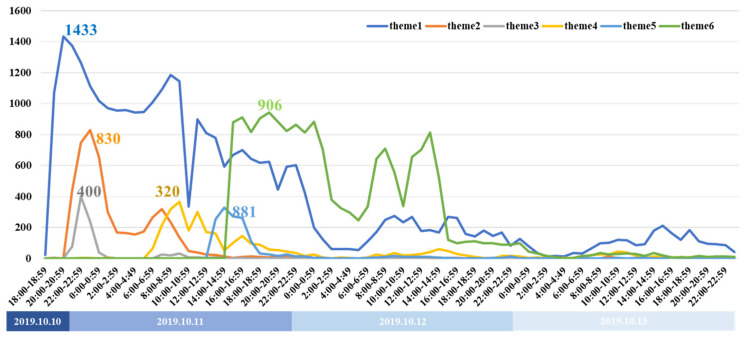
Number of messages related to the accident on different themes.

The change rules for the number of messages in each theme are shown in Figure 7, with the curves of different colors representing the different themes. When a new theme was generated, it often attracted attention quickly. The most obvious changes were in the sharp increases in interest when new themes emerged, which were also accompanied by a rapid decline in the previous theme’s discussions; for example, the emergence of Themes 2 and 3 led to a decline in the discussion of Theme 1, the emergence of Theme 4 was accompanied by a decline in the discussions of Themes 2 and 3, and the emergence of Theme 5 was accompanied by a decline in discussions on Theme 4. Because Theme 6 attracted a great deal of interest, the discussions on the other themes dropped sharply to near zero. In particular, Theme 3 was officially confirmed as a rumor shortly after its appearance, and the theme discussion quickly disappeared thereafter, which somewhat proved the importance of timely rumor removal to curb the spread of rumors.

**Figure 8:**
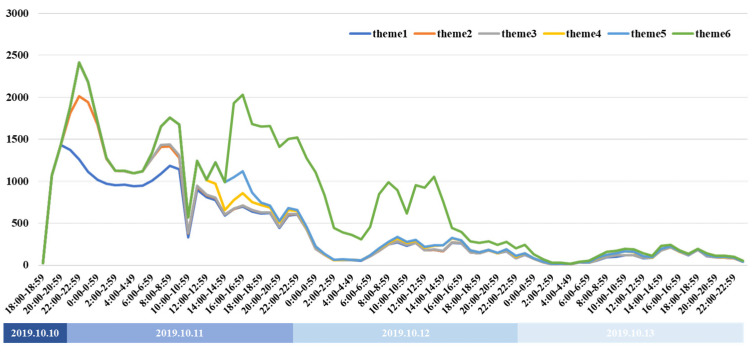
Trends for the six themes for the number of messages related to accident.

As the start time of each theme was different, the change trends in the six themes were simulated in Figure 8, from which it can be seen that the change trends were similar. Therefore, although the sequence of themes was different, the discussion heat in each appeared to have a certain consistency.

### 4.2. Temporal Variations in the Trending Themes

**Figure 9:**
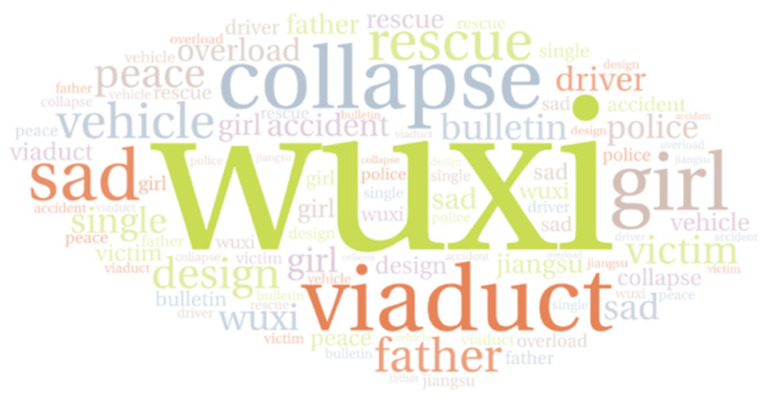
Word cloud for words related to the accident.

**Figure 10:**
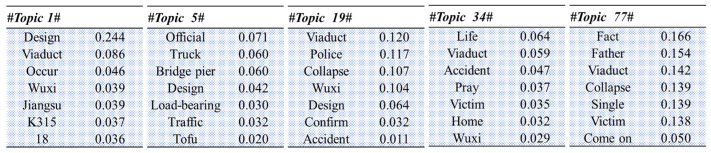
Partial results for the topic analysis.

To analyze the changes after the accident in the Sina-Weibo public opinion focus over time, topic analysis using the LDA model was applied. The data were first segmented to identify the high-frequency vocabulary being used to discuss the accident, which as shown in Figure 9 was basically the same as the topic keywords for the six topics selected using the Sina-Weibo hashtags. All 59,263 messages were divided into 11 equal parts along the timeline; however, a fixed time period was not set because there may have been a large difference in the data quantities in the same time pane. To determine the number of topics in the LDA model for each data set, repeated experiments were conducted to determine the optimal number of topics with the least overlap, with the optimal number of topics in each group being found to be 10 to ensure clear and unique topic features. Therefore, the number of topics in the LDA model was set to 10, with a total of 110 topics identified for the 11 data sets. Some of the unrelated topics were removed, so finally 84 topics related to the accident were identified, some of which are shown in Figure 10. The number after each word represents its probability of occurrence under this topic. Combined with expert opinions, we condensed all topics according to these words, which could be summarized under four main headings: (1) accident information; (2) loss and damages; (3) questioning liability; and (4) emotions. Based on these four topics, we selected three experts to classify eighty-four topics by voting, the results of which are shown in Table 2.

**Figure 11:**
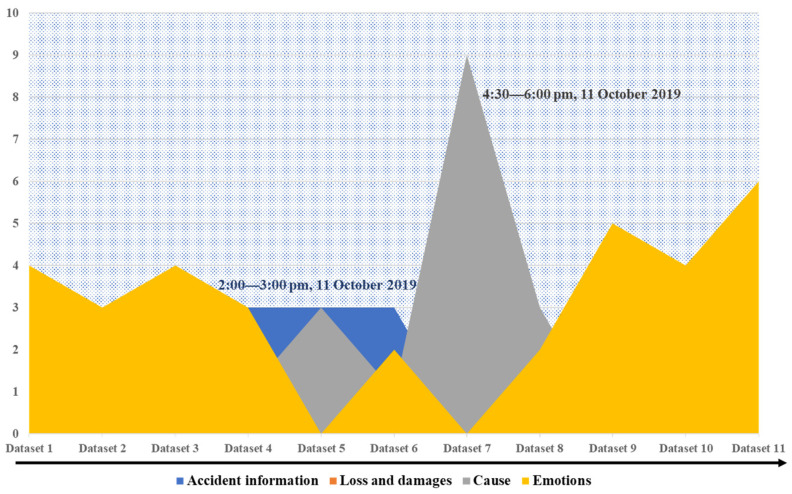
Temporal variations in the topic discussions.

Figure 11 shows the intuitive results for the topic analysis, from which it can be seen that emotional expression played an important role in the public opinion development for the entire accident. Without going through the accident or understanding the accident causes, the social media was a platform for people to express their emotions. Greater attention to “accident information” was found in Dataset 6. The original data indicated that this data set was composed of messages issued by officially certified users from 2:00 p.m. to 3:00 p.m. on 11 October 2019, at which time most users were at work as discussed in Section 4.1; therefore, the main relevant accident information was disclosed by the official media in this period. Dataset 7 was focused on “questioning of liability”, which reached its zenith between 4:30 p.m.–6:00 p.m. on 11 October 2019, at which time the media had revealed that one of the victims had been a single father. Sympathy for the victims inspired the public to question who was responsible for the accident; therefore, dissatisfaction with the responsible party and mourning for the victims were both being expressed.

### 4.3. Temporal Variation of Public Sentiment

**Figure 12:**
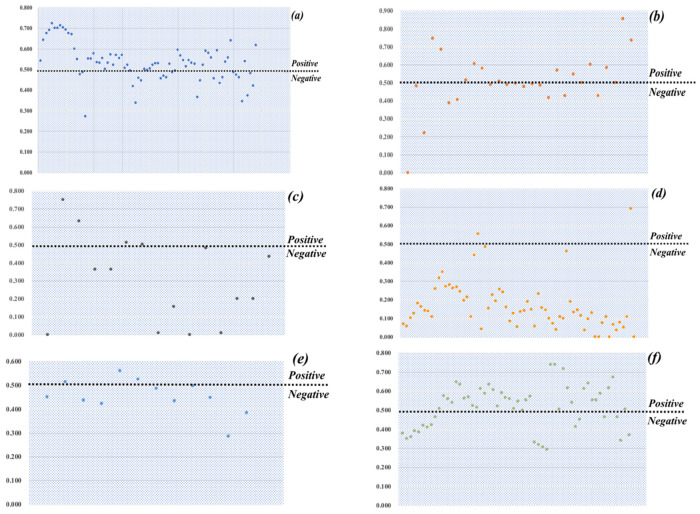
Sentiment analysis for the six themes ((**a**) # Wuxi Viaduct collapse in Jiangsu province #, (**b**) #Wuxi police reported viaduct collapse#, (**c**) # The viaduct was designed by JSTI Co., Ltd.#, (**d**) # 3 dead, 2 injured in Wuxi Viaduct collapse #, (**e**) # The boss of Wuxi Success Transportation Co., Ltd., the vehicle’s owner, was taken away by police #, (**f**)# One of the victims was a single father #).

The BiLSTM algorithm was adopted to score each user message on the different themes to identify the emotional changes in the accident. The average score for a user’s microblog was calculated for the data processing, and the part with too little data discarded. Figure 12 shows the changing user emotions for the six themes, from which different attitudes for different themes can be seen. The highest overall emotional score was for theme six as the accident was distressing and the identity of the single father victim more likely to cause discussion. Under this theme, people expressed condolences and blessings to the deceased as well as sympathy for the little girl who lost her father, which indicated that users were more concerned about the victims than the incident itself.

The lowest overall emotional score was for Theme 4 with the discussion focused on bridge quality, dissatisfaction with Government actions, and distress for the human casualties and economic losses. Similar negative emotions were found for Themes 3 and 5, which discussed the construction unit of the bridge and the perpetrator of the accident. Although Theme 3 was eventually confirmed to be a rumor, users who did not know the truth initially expressed negative emotions during the discussion. Themes 1 and 2 ran through the whole event, and as the users discussed various types of related news under these two themes, the emotional scores fluctuated in concert with the users’ changing perceptions.

### 4.4. Correlations between Social Media Activities and Public Sentiment

To further verify the similarities between the social media activities and the Sina-Weibo platform users’ emotional changes towards the accident, a correlation analysis was conducted on the collected Sina-Weibo messages across eight data dimensions: number of messages/forwards/comments/likes, number of messages posted by official-authenticated/personal-authenticated account/personal accounts, and max number of messages per minute; and the public sentiment score data. Four indicators showed correlation characteristics of sig < 0.05, as shown in Table 3.

The number of messages posted by the personal-authenticated accounts showed the strongest correlation. Although there were many microblogs posted by official users, most were republished official messages released by the Government. The repetitive content was very high and could not have had much influence on user moods. As personal authentication accounts have a certain credibility and many followers, their opinions about such accidents can often affect user emotions. For this accident, the number of Weibo messages and users’ emotions had a positive correlation. The important reason was that the Government played a positive role in this incident by quickly refuting rumors and announcing event progress, which was reflected in the positive sentiment of the users.

### 4.5. Discussion

This study sought to comprehend the evolution of social media user behavior and emotions during emergency events. Different from previous studies, more emphasis was put on the data collection stage and the refinement of the original data. Therefore, unlike previous studies, the endogenous trends in social media activities were not examined; rather, this study took a continuous exposure focus on the same event to examine the impact of related events on the behaviors and emotions of the social media users. By analyzing the content in the different related topics, it was possible to compare the laws of the respective social activities and identify the correlations.

The data text and sentiment analysis connected the overall data and segmented data to elucidate the changes in user behavior and emotional responses and explore the correlations between the popular events and the social media user focus; therefore, future research plans to explore the general law further by tracking more events.

The Weibo messages publishers were carefully examined, and the event developments compared to identify the roles played by these different social media users in response to emergencies and to understand the user behavior in these types of situations. This research could assist in streamlining social media emergency information releases to integrate media resources and ease public anxiety.

A large number of similar events occur every year and there are also many discussions on social platforms, however, the Government deals with different events in different ways. Judging the different dissemination effects of official information released at different time points and determining the different effects of the official information released at different event points on user emotions is important to improve the effectiveness of official information in emergencies.

This paper provides some novel insights on refining social media research data, identifying and extracting data sources, perceiving the impact of focused themes derived from the accident background on user behaviors and sentiment, and exploring the correlations between them.

## 5. Conclusions and Future Research Directions

With the popularity of mobile devices, after major social events, people tend to use social media to browse event information and express their opinions, which has also made social media an important source of research data. This study selected the “2019 Wuxi Viaduct Collapse” as the research object to examine the evolution of social media activities after emergencies and analyze the emotional changes in the user populations.

This study had innovative data acquisition. Data from different themes under the same accident were analyzed to develop a framework to track the evolution of the social media user behavior and public sentiment and analyze their correlations. This analysis found that the social media activities were in line with normal daily activities and that users did not appear to intentionally change their schedules because of the accident. As events developed, new social media themes were generated, which quickly attracted the attention of users and led to an interest decline in the previous theme. However, the trends for the different themes generated under the same event had similar trends.

Research on user concerns found that the most important function of Sina-Weibo for users was to express their feelings and emotions, with the emotions varying significantly depending on the themes. Users were strongly dissatisfied with the damage caused by the accident, and although a specific cause of the accident was not identified, criticisms emerged. However, the users tended to use more positive words for the victims and to express their mourning, and the user sentiments were also positively related to the information released by the personal-authentication accounts, which was because of the large number of followers and their high credibility. The positive correlation with the number of messages and the maximum number of messages per minute suggested that people had a more positive attitude towards what happened at each time node in this event. Another limitation of this paper is that it only analyzes Chinese information, and other users can also discuss emergencies in other languages

There were, however, several limitations to this research. First of all, location information was not applied because there was insufficient geographic data, and the spatial evolution was small after the event. Future research plans to use geospatial data to more closely examine the development of disasters. Second, due to the short duration of this accident, this article only researched the number of messages published by three user types and there was no deep research on the information dissemination network created by different users, which is to be the focus of future research.

## Figures and Tables

**Table 1:** Basic statistics for the accident-related data.

Theme	Time (2019)	No. of Messages	No. of Comments	No. of Forwards	No. of Likes
Theme 1	18:00 10 October–24:00 13 October	31,383	196,595	328,114	4,115,813
Theme 2	21:00 10 October–24:00 13 October	4901	33,638	54,739	940,131
Theme 3	21:00 10 October–24:00 13 October	909	1643	6693	31,428
Theme 4	06:00 10 October–24:00 13 October	3222	48,951	142,368	1,047,056
Theme 5	12:00 10 October–24:00 13 October	1518	1099	4306	33,865
Theme 6	16:00 10 October–24:00 13 October	17,330	6734	4603	23,559
Total	18:00 10 October–24:00 13 October	59,263	288,660	540,823	6,191,852

**Table 2:** Topic classification results.

	Accident Information	Loss and Damages	Questioning of Liability	Emotions
Dataset 1	2	0	3	4
Dataset 2	1	1	2	3
Dataset 3	3	0	3	4
Dataset 4	3	2	1	3
Dataset 5	3	0	3	0
Dataset 6	3	0	1	2
Dataset 7	0	0	9	0
Dataset 8	1	0	3	2
Dataset 9	0	2	0	5
Dataset 10	0	3	0	4
Dataset 11	0	2	0	6
Total (percentage)	16(19%)	10(12%)	25(30%)	33(39%)

**Table 3:** Correlation coefficients between public sentiment and the number of messages, personal-authentication accounts, personal accounts, and maximum number of messages per minute.

		Number of Messages	Personal-Authenticated Accounts	Personal Accounts	Maximum Number of Messages per Minute
**Sentiment**	P	0.258	0.433	0.277	0.398
	Sig.	0.023	0.000	0.014	0.000

## Data Availability

By examing the event timelines and the associated hashtags on the popular Chinese social media site Sina-Weibo (https://weibo.com/login.php accessed on 20 October 2019). The 2019 Wuxi viaduct collapse accident was taken as the research object and the event timeline and the Sina-Weibo tagging function focused on to analyze the behaviors and emotional changes in the social media users and elucidate the correlations.
